# Spatio-temporal patterns, trends, and oceanographic drivers of whale shark strandings in Indonesia

**DOI:** 10.1038/s41598-025-20543-3

**Published:** 2025-10-17

**Authors:** Mochamad Iqbal Herwata Putra, Anindya Wirasatriya, Haidar Asyraffauzan, Fahmi, Ismail Syakurachman, Abdi Hasan, Hanggar Prasetio, Abraham Sianipar, Edy Setyawan, Prabowo Prabowo, Muhammad Subhan Wattiheluw, Arief Edy Handoyo, Muhammad Firdaus Agung Kunto Kurniawan, Mark V. Erdmann, Jatna Supriatna, Masita Dwi Mandini Manessa

**Affiliations:** 1 Department of Geography, Faculty of Mathematics and Natural Sciences, Universitas Indonesia, Depok, Indonesia; 2 Focal Species Conservation Program, Ocean and Science Department, Konservasi Indonesia, Jakarta, Jakarta Indonesia; 3 Department of Oceanography, Faculty of Fisheries and Marine Science, Universitas Diponegoro, Semarang, Indonesia; 4 Center for coastal Rehabilitation and Disaster Mitigation Studies, Universitas Diponegoro, Semarang, Indonesia; 5 Research Center for Oceanography, National Research and Innovation Agency, Jakarta, Indonesia; 6 Department of Biology, Faculty of Mathematics and Natural Sciences, Universitas Indonesia, Depok, Indonesia; 7 Elasmobranch Institute Indonesia, Denpasar, Bali, Indonesia; 8 Directorate of Aquatic Biota and Ecosystem Conservation, Directorate General of Marine Spatial and Ocean Management, Ministry of Marine Affairs and Fisheries, Jakarta, Indonesia; 9 Conservation International Asia-Pacific, Auckland, New Zealand; 10 ReShark , Auckland, New Zealand

**Keywords:** Whale shark, Stranding, Upwelling, Hotspot analysis, Indonesia, Ecology, Ocean sciences

## Abstract

Despite the increased international attention to whale shark conservation, their populations remain predominantly depleted due to anthropogenic activities such as fishing, ship collisions, and marine pollution. Reports of whale shark strandings in Indonesia have been increasing in recent years, elevating concerns regarding their well-being and the potential disturbance to their population recovery. However, limited understanding of stranding patterns, trends, and the oceanographic factors potentially driving these events has resulted in efforts focusing primarily on responding to strandings rather than implementing effective mitigation strategies. Using a 13-year stranding dataset (*n* = 115) obtained from open-access databases, reports, news, and publications, we examined the characteristics of stranding cases in Indonesia, including population demographics, where hotspots occur, and whether their occurrence is related to oceanographic dynamics in the region. Our study highlights significant population-level disturbances, with 70% of stranded individuals being large juveniles (4–7 m). It also documented a positive interannual trend in stranding cases (R² = 0.67, *p* < 0.01). The southern coast of Java has emerged as a stranding hotspot, with events seasonally associated with strong upwelling, likely related to the seasonal foraging activities of whale sharks in the region. Although natural events were identified as the main factors contributing to whale shark strandings, anthropogenic activities may also play an important role and require further investigation.

**Supplementary Information:**

The online version contains supplementary material available at 10.1038/s41598-025-20543-3.

## Introduction

 The whale shark (*Rhincodon typus*), the largest living fish in the world, inhabits cosmopolitan tropical and warm temperate waters. Whale sharks are currently listed as Endangered on the IUCN Red List because they have been exposed to targeted fisheries and significant bycatch in areas with high population densities, leading to rapid declines in catch per unit effort (CPUE) measures^1^. It is estimated that their global population has declined by at least 50% in the past three generations (75 years), with the Indo-Pacific subpopulation having experienced a 63% decline and the Atlantic subpopulation declining over 30%^1^.

In 2012, the IUCN developed the Green Status Assessment, which aims to complement the IUCN Red List classification by evaluating status in terms of extinction risk and recovery progress and tracking the success of conservation initiatives. Although there has been increased global attention towards the conservation of whale sharks, their populations remain largely depleted due to past fishing pressure^2^. The recently released IUCN Green Status Assessment for the whale shark emphasizes that although the full recovery of this species might be possible within 100 years, it will require concerted and sustained effort^2^. However, despite the high potential recovery of whale shark populations, the number of natural deaths and stranding events on coastal beaches is also increasing^3^. These challenges in recent years have raised concerns regarding whale shark well-being and the potential disturbance to population recovery.

Whale shark strandings have been reported in many places, including in Florida, USA^4^, India^5–10^, South Africa^11^, the east and west coasts of Australia^12^, Thailand^13^, China^14^, southern Mozambique^15^, Dutch Caribbean Island^16^, southern and northeast Brazil^17,18^, the Philippines^19,20^, the Gulf of California^21^, Khor al-Zubair Iraq^22^, and Indonesia^23–25^. Public interest in this species has progressively escalated since the emergence of social media, particularly as news of whale shark stranding events have become more frequently reported in Indonesia^26^. Frequent questions from the public, scientists, and authorities regarding the stranding news include curiosity about the reasons behind these large animals getting stranded and ways to assist them^26^.

While the causes of most stranding events are still unknown, a few studies have identified some potential causes of whale shark stranding events. Necropsies on dead whale sharks reported stranded in Brazil, Thailand, and the Philippines revealed that plastic discovered in their stomachs was the most likely cause of death^13,17,19,20^. In several countries like India, Mexico, and Indonesia, interactions between whale sharks and fishing gear (e.g., drift gillnets) have contributed to injuries, stress, and fatigue, aggravating their vulnerability to stranding and mortality from asphyxia^3,8,25^. Finally, environmental factors like sudden changes in water temperature^11^ or proximity between shallow bays and steep continental shelves may disorient or trap whale sharks, making it challenging for them to navigate and return to deeper waters^11,22^, and may also result in strandings.

In Indonesia, most stranded whale shark cases were initially reported as animals swimming close to shore, but not actually stranded on the beach^23,25^. In most cases, despite being alive, the weakened whale sharks were unable to return to an appropriate depth and were often dragged to the shore by well-meaning villagers, resulting in their deaths. Unlike marine mammals, whale sharks cannot survive out of water, hindering rescue efforts^19^. Limited understanding of whale shark biology by coastal villagers, and often times a desire to exploit the stranded sharks, pose challenges to successful rescue operations.

Overall, managing stranded whale sharks poses human resources, logistics, and time challenges. To improve our stranding response management and develop effective mitigation strategies, we require an in-depth examination of whale shark strandings in Indonesia. Using a 13-year data set, this is the first study that evaluates population demographics, identifies where stranding hotspots occur and examines whether their occurrence is related to oceanographic dynamics in the region, in order to develop effective mitigation strategies.

## Results

### Whale shark strandings

**Fig. 1:**
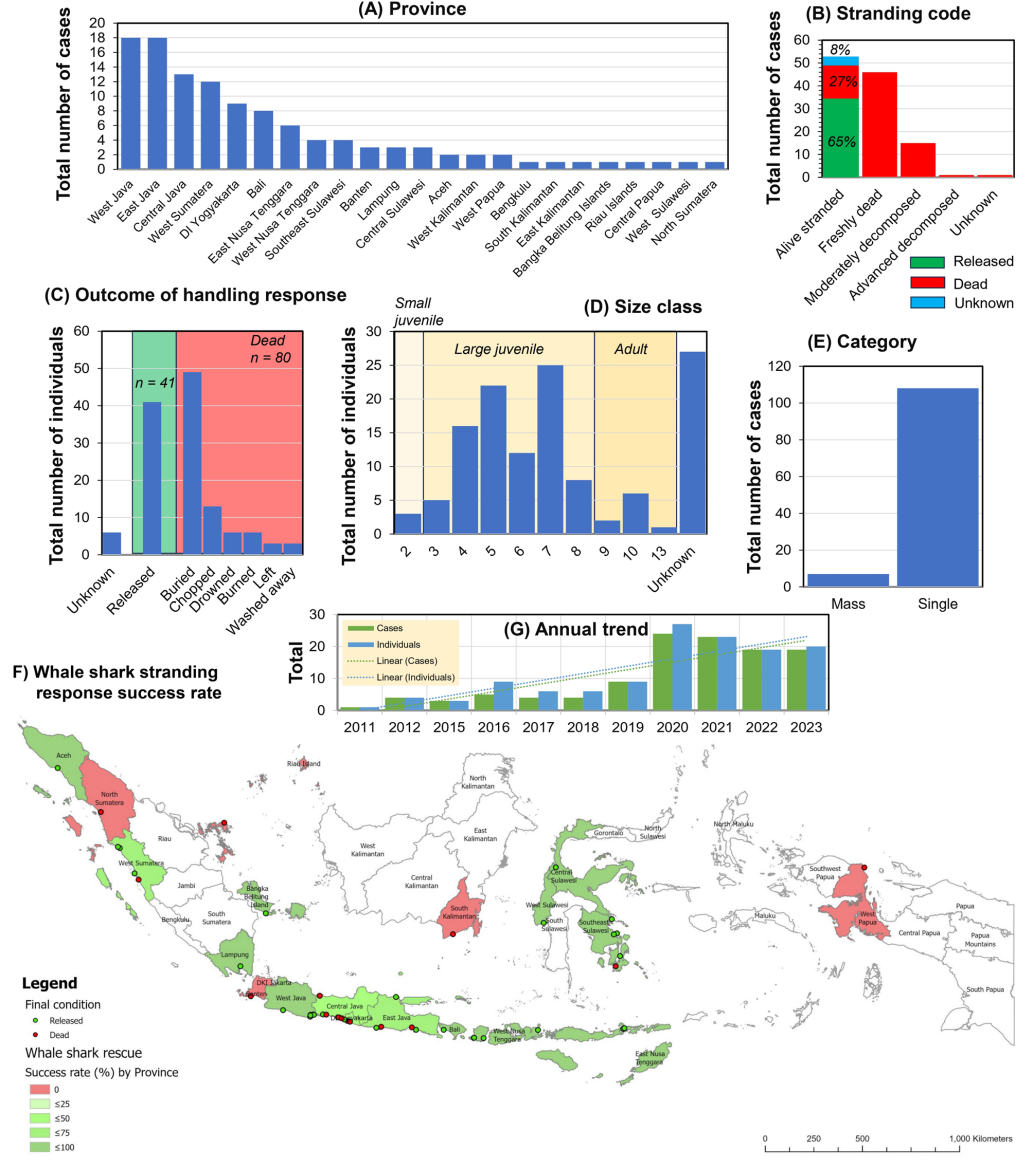
The number and distribution of whale shark stranding cases by province (**A**), condition (**B**), category (**C**), outcome of handling response (**D**), size class distribution (x-axis denotes total length TL in meters, with 3 size classes indicated of small juvenile (< 3 m), large juvenile (3–9 m), and adult (> 9 m); UNK: Unknown (**E**), success rate (summarized by province) of attempted stranding rescues of alive-stranded whale sharks (**F**), and annual trends (**G**).

Between 2011 and 2023, 115 whale shark stranding events involving 127 individuals were recorded across 23 out of 38 provinces in Indonesia (Fig. 1A and Supplementary Figure 1). The highest cumulative number of cases occurred in West Java (total of 18 cases, mean ± SE, 1.64 ± 0.74 cases per year) and East Java (18 cases, 1.64 ± 0.72 cases per year). A Kruskal-Wallis H test revealed a significant difference in whale shark stranding cases among provinces (*χ*^*2*^ = 41.41, df = 22, *p* < 0.05).

The most common stranding code reported was an “alive stranded” whale shark (52 cases, 4.73 ± 1.30 cases per year; Fig. 1B), followed by a freshly dead stranded whale shark (46 cases, 4.18 ± 1.44 cases). These two stranding codes were far more common than the other two codes (i.e., moderately decomposed and advanced decomposed) when whale sharks were discovered (*χ*^*2*^ = 25.67, df = 4, *p* < 0.001). West Java had the highest number of alive-stranded whale sharks (9 cases; Supplementary Fig. 2), followed by Central Java (6 cases) and East Java (5 cases). Meanwhile, East Java had the most freshly dead stranded whale sharks (11 cases), followed by West Java (7 cases) and Central Java (6 cases).

The most common outcomes to whale shark stranding responses were “buried” (49 individuals; 4.45 ± 1.43 individuals per year; Fig. 1C), followed by “released” (41 individuals, 3.73 ± 1.28 individuals per year). These two response outcome categories were the most prevalent (*χ*^*2*^ = 19.74, df = 7, *p* < 0.05) compared to other outcomes (i.e., chopped, drowned, burned, left, washed away). East Java had the highest number of stranded whale sharks managed through burial (Supplementary Fig. 3), with ten individuals, followed by Central Java with nine and West Sumatera with seven cases. West Java, on the other hand, had the highest number of stranded whale sharks released into the wild, with seven individuals, followed by South Sulawesi with five individuals.

The predominant size category for stranded whale sharks is size class seven (≥ 7 - <8 m), with an average of 2.27 ± 0.54 individuals per year and a total of 25 individuals (Fig. 1D). The size class with the lowest frequency recorded is class 13 (≥ 13 - <14 m), with only one such large adult individual reported stranded from Pantai Pandansimo Baru in Yogyakarta. A significant difference in size class was observed among stranding cases (*χ*^2^ = 27.26, df = 10, *p* < 0.05). The region with the highest number of stranded whale sharks in size class seven was East Nusa Tenggara (Supplementary Fig. 4). Among different life stages, large juveniles (≥ 3 to < 9 m) had the highest average stranding frequency at 8.00 ± 2.11 individuals per year (*χ*^2^ = 15.53, df = 2, *p* < 0.01), which was significantly greater than adults (> 9 m, 0.81 ± 0.30 individuals per year) and small juveniles (≥ 1.5 to < 3 m, 0.27 ± 0.14 individuals per year).

Over the study period, single strandings (108 cases) of whale sharks in Indonesia were much higher than mass strandings (7 cases), with this difference statistically significant (Mann Whitney U test, *Z* =−3.71, *U* = 4.5, *p* < 0.001; Fig. 1E). The annual mean of single whale shark strandings was 9.82 ± 2.59 cases, while the annual mean for mass strandings was 0.64 ± 0.28 cases. Aceh, West Sulawesi, and West Sumatera all experienced mass strandings, while East Java and East Nusa Tenggara observed mass strandings twice in each province. The largest mass stranding case occurred in West Sulawesi (Supplementary Fig. 5), specifically at Mampie Beach, with five whale sharks reported stranded on August 31 st, 2016.

Many whale shark strandings resulted in mortality, averaging 7.27 ± 1.64 individuals per year (Fig. 1C). The highest mortality rates occurred in 2022 and 2023, with 15 individuals reported dead each year. A comparison of annual mortality from stranding events and the number of individuals successfully released into the waters (7.27 ± 1.28) revealed no significant difference (*Z* = 1.71, *U* = 87, *p* = 0.087). The general success rate for releasing stranded whale sharks into the wild stands at 71%, with 34 out of 48 cases proving successful (Fig. 1B). West Java was identified as the province with the highest number of stranded whale sharks successfully released alive (9 individuals) - a commendable rescue success rate of 78% (Fig. 1F). Meanwhile the success rate for stranded whale sharks that were released back to the sea in Central Java is 33% and East Java 75%.

### Interannual trends of whale shark stranding

A linear regression model revealed a positive relationship in Indonesia’s whale shark stranding cases (R^2^ = 0.67, *p* < 0.01; Fig. 1G), indicating that the variable “year” explains 67% of the variability in these cases. Between 2011 and 2023, the annual increase in whale shark stranding cases was predicted to be 1.87-fold [95% CI: 0.89, 2.85]. The linear regression model also showed a positive association for individual whale shark strandings in Indonesia (R^2^ = 0.68, *p* < 0.05), highlighting that variable “year” accounts for 68% of the variability in stranded whale shark individuals. Projections showed an annual rise of 1.9 individuals [95% CI: 0.92, 2.89]. The ANOVA test indicated a significant difference in the number of reported stranding events (F = 108.44, η^2^ = 0.92, df = 1, *p* < 0.0001) between the pre-2020 period (4.2 ± 2.43 cases) and from 2020 onwards (22.25 ± 3.30 cases).

### Spatial and Temporal hotspots

**Fig. 2:**
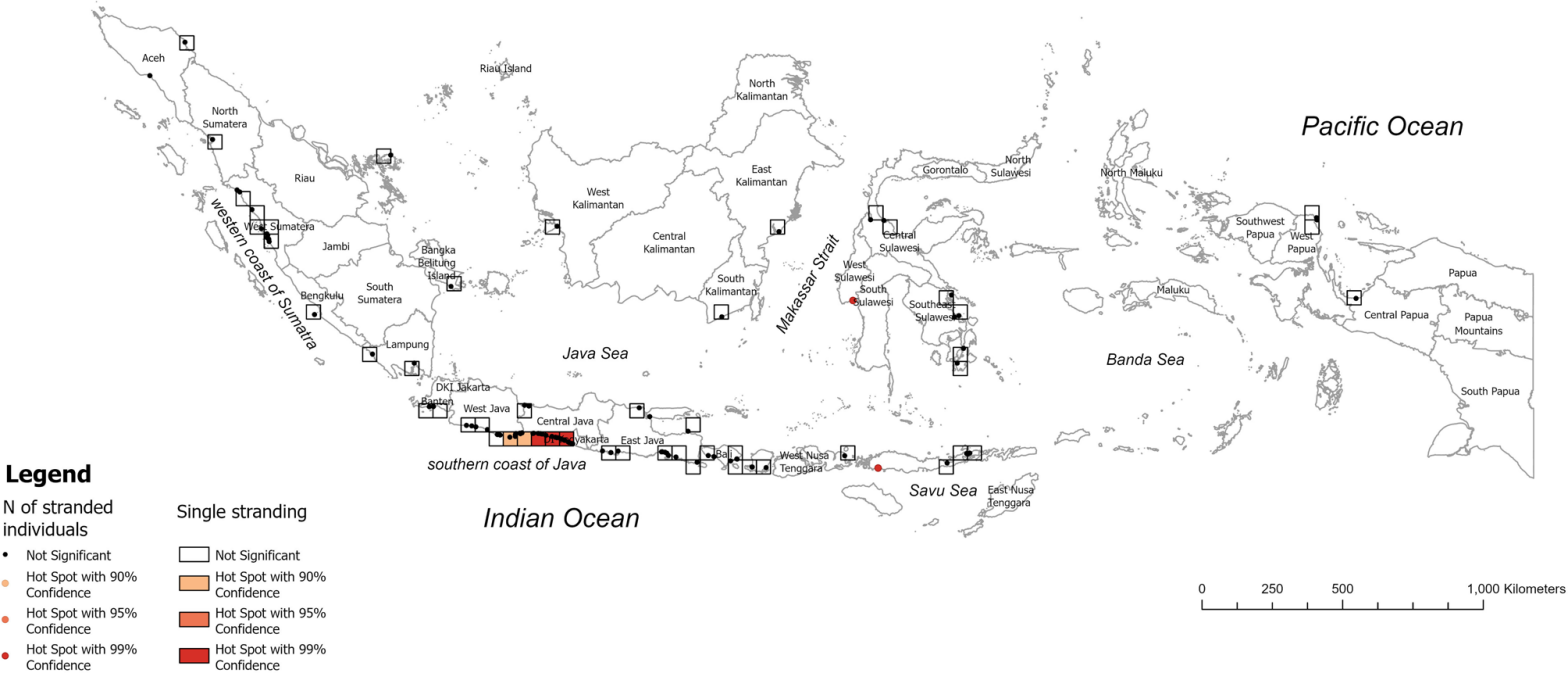
The spatial hotspots analysis of single and individual strandings events of 2011-2023.

Optimized hotspot analysis of whale shark strandings from 2011 to 2023 revealed that a single stranding hotspot occurred on the southern coast of Java (Fig. 2). Central Java had the highest Gi*Z score at 4.40 and 4.86 (*p* < 0.0001, CI = 99%, respectively), followed by DI Yogyakarta (Gi*Z score = 4.20, *p* < 0.0001, CI = 99%). Meanwhile, the southeast coast of West Java was identified as a hotspot with a Gi*Z score of 2.78 (*p* < 0.01, CI = 90%). Furthermore, West Sulawesi was identified as a mass stranding hotspot, as indicated by its Gi*Z score of 8.08 (*p* < 0.05, CI = 99%), followed by East Nusa Tenggara with Gi*Z score of 5.58 (*p* < 0.05, CI = 99%).

**Fig. 3:**
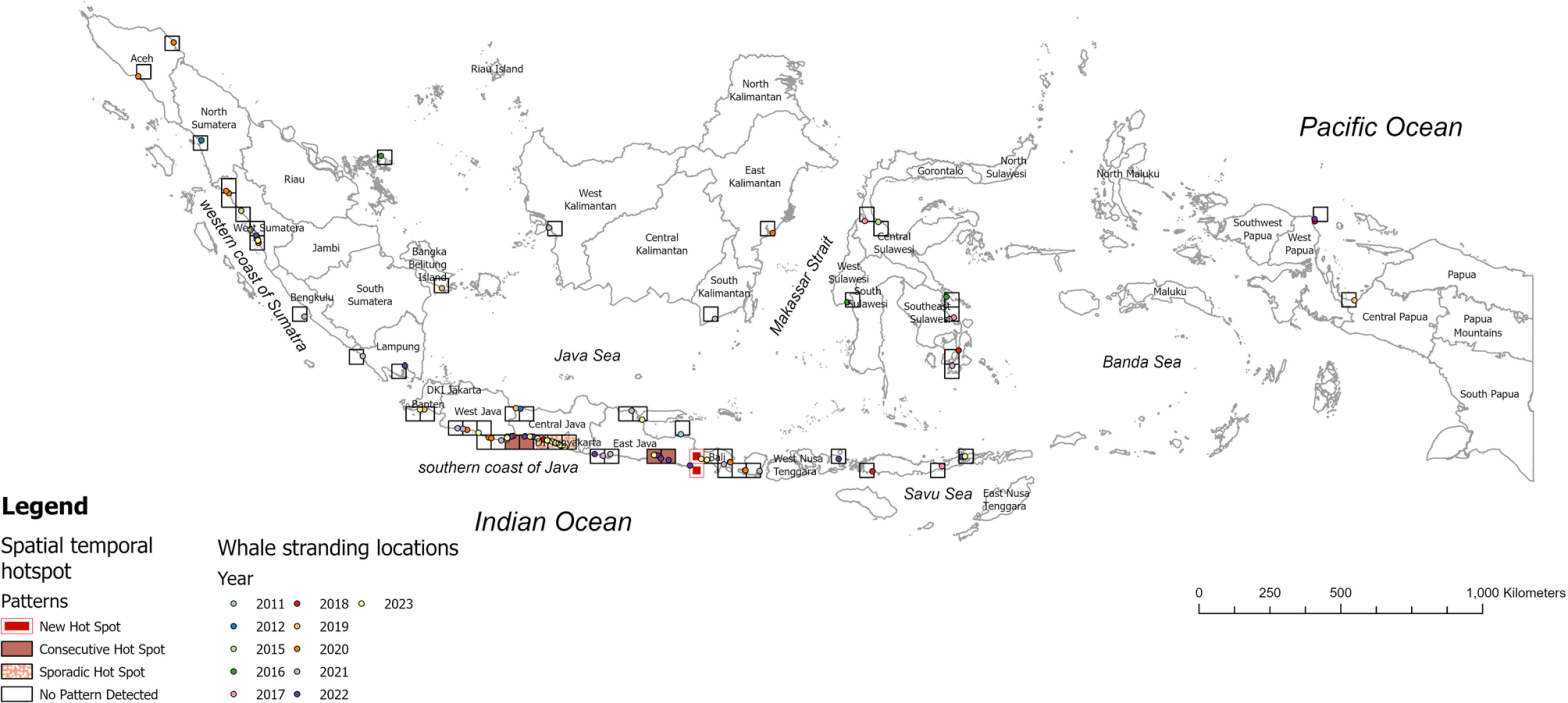
The patterns of spatial-temporal hotspots and whale shark stranding locations recorded between 2011 and 2023.

Nine out of 57 locations have a space-time hotspot pattern, which includes consecutive hotspots, sporadic hotspots, and new hotspots. Consecutive hotspots were detected extending from Pangandaran in West Java to Karang Pakis Beach in Cilacap, Central Java, and from Selok Anyar to Cangakan Beach in East Java. These locations exhibited a single uninterrupted run of at least two statistically significant hotspot locations in the final time-step in 2023, without prior significance being hotspots (Fig. 3). Sporadic hotspots were identified from Banjasari Village Beach in Cilacap Regency, Central Java, to Parangtritis in Yogyakarta. These areas were statistically significant hotspots in the final time step of 2023. Still, they fluctuated as hotspots for whale shark strandings throughout the study period, with no occurrences of statistically significant cold spots. Furthermore, Ngagelan Beach in Banyuwangi (East Java) and Yeh Kuning Beach in Jembrana Bali have emerged as new hotspots. This indicates that the area had never previously been identified as a stranding hotspot. However, the most recent time step (2022–2023) shows statistically significant hotspot activity, marking it as a newly emerging hotspot.

Analysis of annual trends reveals that multiple sites along the west coast of Sumatra Island, including Tan sirdano, Taluak Batuang, Surantih, Ulak Karang, Kincia, Sungai Pampan, and Sungai Pinang beaches, have experienced increasing stranding events (Tren Z = 2.28, *p* < 0.05, CI = 95%). The Pandan beach in Central Tapanuli Regency was the only area experiencing a downward trend on the west coast of Sumatra Island (Tren Z = −2.26, *p* < 0.05, CI = 95%), with the last stranding case in 2012. Many areas in the southern coast of Java experienced an increase in stranding events over the course of our 13-year study (CI = 90–95%), with the highest hot spot locations occurring from Kemiren Cilacap and Karang Pakis in Central Java (Tren Z = 2.82, *p* < 0.001). Semiring village beach in Situbondo (East Java) was the only area on Java experiencing a downward trend (Tren Z = −2.16, *p* < 0.05, CI = 95%), with the last stranding happening in 2011. In Bali, the Jembrana coast (Yeh Kuning Beach) shows the most significant increase in stranding incidents (Tren Z = 2.12, *p* < 0.05, CI = 95%). Lastly, East Flores Regencies in Nusa Tenggara Islands, also exhibited an upward trend in stranding incidents (Tren Z = 2.12, *p* < 0.05, CI = 95%).

### Influence of seasonal oceanographic dynamics

Cumulatively, the seasonal distribution of whale shark stranding cases was higher during seasonal transition II (Sep-Nov), with a total of 53 cases, followed by southeast monsoon (SEM) with 30 cases. Central Java had the most cases (10 cases) during seasonal transition II in the past six years of the study period. East Java and West Java, which have recorded the highest number of whale shark strandings, reported elevated cases during the southeast monsoon (10 cases) and seasonal transition II (9 cases), respectively (Supplementary Fig. 6).

The Hovmöller plot of daily climatology showed that upwelling begins in late May, marked by increased chlorophyll-a concentrations, higher wave heights, and cooler sea surface temperatures moving from the eastern region of Indonesia (Savu Sea at Lesser Sunda) to the southern coast of Java, and ending in the west coast of Sumatra (Fig. 5A, D, G). In January to March, wave height only slightly increases, impacted by the peak of westerly wind during the Northwest monsoon season. Climatology Hovmöller plots indicate that many whale shark stranding events occur during the upwelling period. On the southern coast of Java, strong upwelling from July to October coincides with numerous whale shark stranding events in this area (Fig. 5B, E, H). Despite no upwelling signal on the western coast of Sumatra from June to September and on the southern coast of Java from January to March, some whale sharks strandings were also observed, possibly due largely to the high waves recorded at this time of year.

**Fig. 4:**
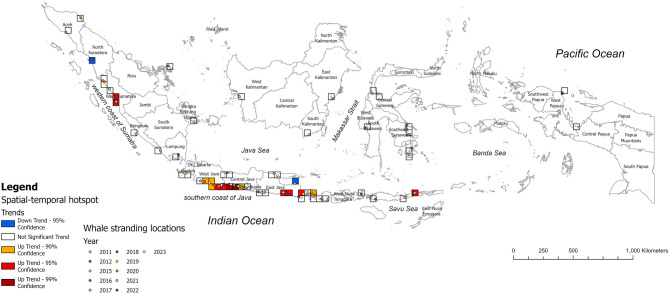
Trends in spatial-temporal hotspots and whale shark stranding locations recorded between 2011 and 2023.

**Fig. 5:**
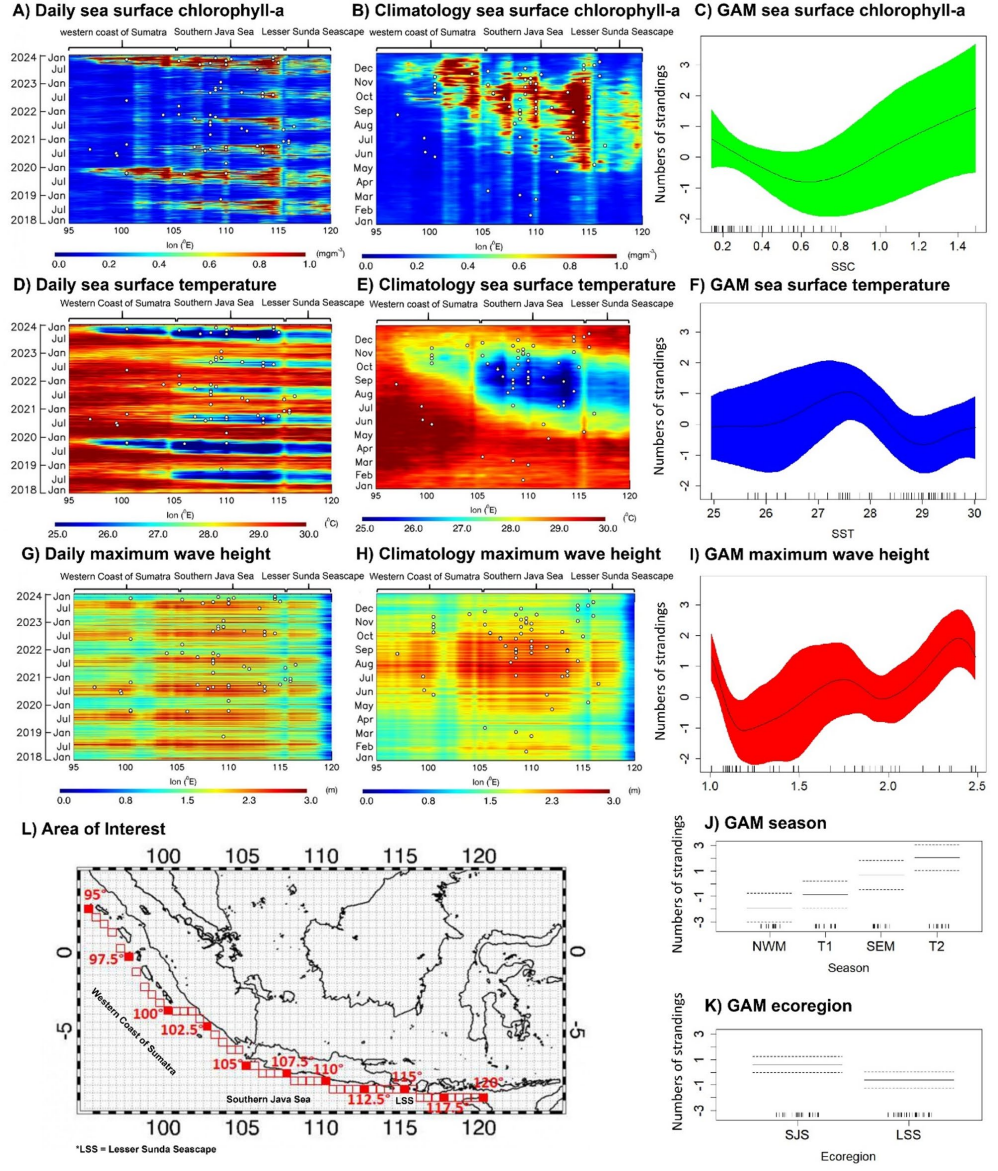
Hovmöller plots of daily and monthly climatology oceanographic variables from 2018 to 2023 that are associated with whale shark stranding events. White dots denote whale shark stranding events. The Negative Binomial Generalized Adaptive Model curve illustrates the influence of each predictor’s variability on the number of whale shark stranding events.

The Negative Binomial Generalized Additive Model (NB-GAM) of season and ecoregion (Southern Java Seascape [SJS] and Lesser Sunda Seascape [LSS]) further confirmed a strong seasonal pattern, with a significant increase during the SEM and seasonal transition II periods (*X*² = 29.83, *p* < 0.0001, df = 3). There was a higher number of stranding events in the SJS ecoregion compared to the LSS (*X*² = 4.57, *p* < 0.05, df = 1). This model explained up to 51.8% (R^2^ = 0.13) of the observed deviation and had an AIC score of 159.74 (Fig. 5J-K).

Seasonal factors influencing whale shark strandings in the SJS and LSS appear to be dominated by oceanographic conditions. This model effectively explains the stranding patterns of whale sharks based on sea surface chlorophyll-a (SSC), sea surface temperature (SST), and maximum wave height (MWH), accounting for up to 63.9% of the deviance explained (R² = 0.80) with an AIC score of 147.71. The NB-GAM identifies MWH as the most significant predictor (*X*² = 53.17, *p* < 0.0001, df = 8), followed by season (*X*² = 7.18, *p* < 0.01, df = 3), SST (*X*² = 13.94, *p* < 0.05, df = 8), and SSC (*X*² = 9.136, *p* < 0.05, df = 8). The NB-GAM curves illustrate that whale shark stranding incidents tend to occur when SSC concentrations exceed 1 mg/m³ (Fig. 5C), SST ranges between 26 and 28 °C (Fig. 5F), and MWH exceeds 1.5 m (Fig. 5I), all of which are characteristics of upwelling conditions.

## Discussion

Effective conservation of highly migratory species, which cover vast areas or are rarely seen, is often impeded by a lack of comprehensive data on their ecology, life history, abundance, distribution, and population trends^27^. Long-term opportunistic records of strandings can be a valuable resource, providing ecological insights into species distribution^26,28^, demographics^29^, and population structure^30,31^, primarily when covering broad spatial and temporal scales^32–35^. This study is the first in the Indo-Pacific region that provides valuable insights into the population demographics of whale sharks stranded over a 13-year period, along with stranding patterns, trends, and hotspot locations influenced by oceanographic conditions.

This study highlights that Indonesia alone recorded 115 whale shark strandings, surpassing the previously reported global combined total of 114 strandings^36^. In that global report on shark strandings^36^, most strandings were documented from South Africa (45 records) and Mexico (29 records). That earlier study focused on sources from scientific literature in indexed databases such as Scopus and Web of Science, as well as grey literature, iNaturalist, and social media platforms, using only English-language keywords. In contrast, our method utilized the Google search engine with a combination of national (i.e., Bahasa Indonesia), and English keywords, substantially improving data collection. We further emphasize the importance of incorporating local media sources, as stranding events involving large and charismatic marine megafauna often attract widespread attention from both national and local journalists.

However, we acknowledge that relying on data collected through community reporting, particularly from the internet and social media, may introduce reporting biases. A significant finding is the notable increase in reported strandings from 2020 onward, likely driven by greater awareness of whale shark conservation, leading to more active community reporting^26^. Additionally, data sourced from community members or non-researchers has its limitations, as it often lacks biological details such as sex and proper photo identification, which are crucial for understanding the population structure^37^ and connectivity of whale sharks with other aggregation sites^38^. Despite these limitations, our study highlights emerging challenges for whale shark conservation in Indonesia, particularly the prevalence of juvenile strandings, which poses critical risks to long-term population recovery that could take up to a century^2^. While natural factors like seasonal upwelling in the southern coast of Java is shown here to contribute significantly to strandings in identified hotspots, human activities such as ship strikes^39^, marine pollution^40^, and fishing practices^25^ may also play a significant role, requiring further investigation.

Indonesia has been recognized as a critical coastal constellation region for juvenile whale sharks, serving as an essential site for key life history such as feeding and movement^41,42^, making it essential to ensure the survival of these juvenile sharks to rebuild healthy populations in the Indo-Pacific subpopulation. Unfortunately, over 70% of reported whale shark strandings in Indonesia involve large juveniles, with most ranging between 4 and 7 m. Numerous other reports also document the majority of whale shark strandings involve juveniles^12,13,17,20–22^. Small and large juvenile males dominate the size segregation of whale shark populations in coastal areas like Indonesia^37,43–50^, which utilize these nutrient-rich environments to fulfil their energetic needs for rapid growth^51,52^. Once they reach reproductive maturity, typically at 7–9 m in total length “TL” (depending on sex and geographic region), whale sharks transition to an offshore lifestyle, likely to exploit offshore prey or for breeding purposes, or a combination of both^52^. Therefore, sightings of adult (> 9 m TL) and female whale sharks in the region are scarce^47,50^^,^^53^ in concordance with our observation that most stranded whale sharks in the region are juvenile males.

This population disruption to juvenile whale sharks poses a serious threat to their potential recovery from previous fishing pressures, which have already reduced the Indo-Pacific subpopulation by more than half^1^. Over the past decade, our study documented 80 whale shark mortalities, with 64 identified as juveniles. These numbers are substantial when compared to the population sizes of whale shark constellations in key regions of Indonesia and surrounding areas, such as Cenderawasih Bay (159 individuals)^42^, Kaimana (95 individuals)^42^, Saleh Bay (108 individuals)^54^, Gorontalo (43 individuals)^55^, Derawan (23 individuals)^56^, Talisayan (75 individuals)^56^, Christmas Island (45 individuals)^56^, and Ningaloo Reef (1,473 individuals)^56^. While this study does not provide direct evidence through photographic identification linking the deceased whale sharks to these populations, movement tracking based on satellite tags suggests that whale sharks from Ningaloo Reef^57–59^, Christmas Island^60^, and Saleh Bay^39,54^ visited areas in the southern coast of Java (Supplementary Fig. 7A), which was identified as a stranding hotspot in Indonesia. Further research is necessary to validate this hypothesis. The only concrete evidence linking a stranded whale shark in Indonesia to a known constellation is a tagged whale shark from Cenderawasih Bay that stranded and died in the same region 13 months after tagging. This whale shark stranded on September 25th, 2019, near Kampung Wanggar in Nabire, and appears to have been chasing anchovy into shallow waters during a period of high productivity, leading to its stranding (Supplementary Fig. 7B).

Although whale sharks are primarily solitary animals, they are also known to form predictable seasonal aggregations at over 20 locations worldwide^38^. These aggregations are driven by the patchy distribution of prey and generally limited food availability in oligotrophic waters like Indonesia, encouraging whale sharks to search for areas with higher productivity^61^. Such aggregations are often linked to upwelling events characterized by high productivity, strong currents, and cooler temperatures^62^. In Mexico’s Caribbean waters, recognized as one of the most important sites for whale sharks, aggregations of over 300 individuals have been observed at a time, driven by a zooplankton bloom occurring during the upwelling period from June to October^63^. These patterns are often predictable due to the recurrence of seasonal upwelling events and increased water productivity in specific regions^64^.

**Fig. 6:**
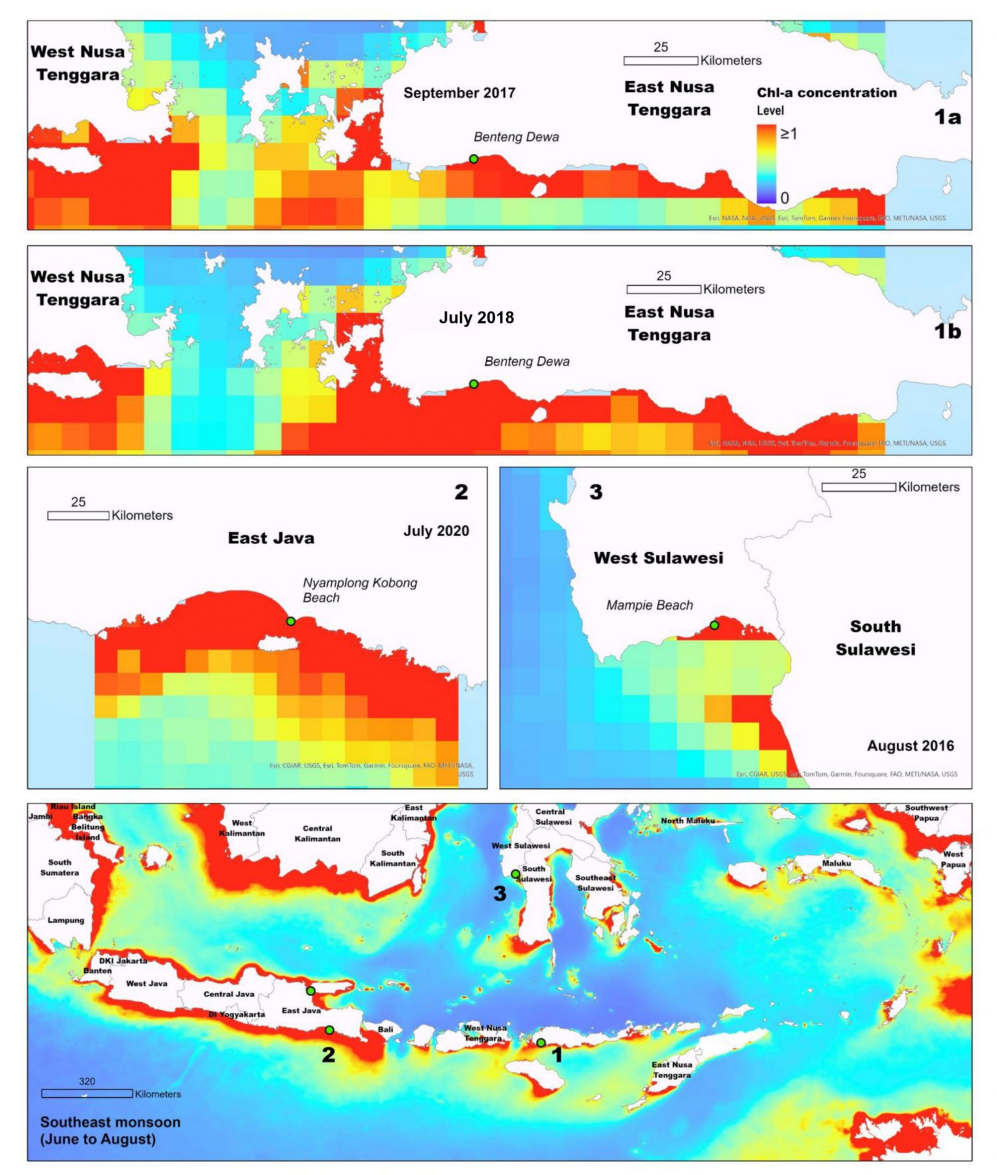
Spatial association between mass whale shark stranding events and upwelling patterns. Sea surface chlorophyll-a concentrations indicate seasonal upwelling intensity, with the bottom panel showing the general trend during the Southeast Monsoon and seasonal transition II. Insets highlight specific regions; green dots mark stranding sites with corresponding dates (e.g., Benteng Dewa –July 2018). The spatial overlap suggests strandings may occur when whale sharks approach coastal areas to feed during high productivity periods.

Our study revealed that mass strandings of whale sharks on Indonesian coasts are often associated with upwelling events. For instance (Fig. 6), a mass stranding of five whale sharks at Mampie Beach in West Sulawesi coincided with a period of wind-driven currents in a region with complex seabed topography in the Makassar Strait, which led to upwelling^65^. In East Nusa Tenggara, two separate mass strandings of three whale sharks occurred in September 2017 and July 2018, aligning with southeast monsoon winds from Australia that induced seasonal coastal upwelling in the region^66^. These upwelling events likely attract whale sharks to these areas as they pursue krill or small fish, such as anchovies, into shallow waters, increasing their risk of stranding. Similar behaviors have been observed in other locations, such as in Iraq^22^ and the Dutch Caribbean region^16^. Moreover, cold-stress-related mortality has been documented in marine megafauna due to sudden upwelling-driven cooling. A recent study found that abrupt temperature drops along major boundary currents, such as the Agulhas and East Australian Currents, can lead to marine megafauna deaths^67^. This is relevant to our findings, which also highlight whale shark strandings during strong upwelling periods along the southern coast of Java, suggesting that abrupt thermal changes, alongside prey concentration, may contribute to stranding risk.

The high waves during the upwelling period along the west coast of Sumatra and the southern coasts of Java and Nusa Tenggara can worsen the situation for whale sharks trapped in shallow waters by continuously pushing them toward the shore, eventually leading to exhaustion and death. Similar occurrences have been reported in South Africa^11^, where whale shark strandings are believed to result from rapid changes in water temperature that slow their metabolic activities, and/or the combination of heavy wave activity and a steeply sloped continental shelf pushing sharks ashore.

Our study indicates that both single and mass whale shark stranding events along the southern coast of Java and Nusa Tenggara are strongly associated with upwelling phenomena during the SEM and seasonal transition II (from June to November). Both daily and climatological Hovmöller plots reveal that many whale shark strandings occurred during this upwelling period. The strong upwellings on the southern coast of Java align with a high frequency of whale shark strandings in the region. According to Wirasatriya et al. (2020)^68^, upwelling along the southern coast of Java occurs under the increasing easterly wind that strengthens the offshore Ekman transport and Ekman suction. The strong easterly wind not only increases upwelling intensity, but also increases the wave height during the southeast monsoon season (Fig. 5H). This seasonal pattern highlights a significant correlation between coastal upwelling and whale shark strandings, emphasizing its critical role in driving their movement and increasing their vulnerability to stranding in these areas. This may help explain why the southern coast of Java, encompassing the provinces of West Java, Central Java, Yogyakarta, and East Java, emerged as a consecutive and sporadic hotspot for stranding events (Fig. 3).

Daily Hovmöller plots from 2018 to 2023 indicate the interannual variation of upwelling (Fig. 5A, D). The interannual variation of upwelling in the study area is strongly influenced by the El Niño Southern Oscillation (ENSO) and Indian Ocean Dipole (IOD), as indicated by the Oceanic Niño Index (ONI) and Dipole Mode Index (DMI) (Supplementary Fig. 8). The strong upwelling in 2019 and 2023, which is denoted by maximum chlorophyll-a concentrations and minimum SST, corresponded to the strong positive IOD. Moreover, the strong positive IOD in 2023 also aligned with strong El Niño conditions. In contrast, the weak upwelling in 2020 to 2022 corresponded to the La Niña and negative IOD. All these results were in accordance with Wirasatriya et al. (2020^68^, 2021^69^). Nevertheless, the pattern of whale shark stranding did not follow the interannual signal of upwellings, suggesting the variation in annual whale shark strandings may correspond more to wave height during the SEM, which does not generally show much interannual variation.

Despite the strong seasonal correlation between whale shark strandings and upwelling phenomena, human-induced factors such as fishing, ship strikes, and marine pollution likely contribute significantly to these events. Gillnet fishing activities along the southern coast of Java, which typically peak during the upwelling season, target the large numbers of pelagic fish that tend to aggregate in coastal areas^70^. Unfortunately, whale sharks are also likely chasing the same prey, leading to a high risk of bycatch, as reported by Nijman et al. (2023)^25^. Additionally, the high risk of ship strikes on whale sharks on the southern coast of Java has been predicted by Womersley et al. (2022)^39^. While there is currently no direct evidence linking documented strandings in this study to fatal injuries caused by ship strikes, this is likely due to the limited number of necropsies conducted to determine the cause of death. The only reported necropsy performed on whale sharks stranded in Indonesia involved two individuals stranded in Kebumen, Central Java, in 2023^40^. A histopathological analysis of their kidney, liver, heart, and intestinal tissues revealed fatty degeneration in the liver and kidney necrosis, likely caused by exposure to toxic substances in coastal environments. The study also found evidence of eutrophication near the stranding sites, which might be associated with waste from aquaculture ponds, particularly uneaten shrimp feed. These three human-induced factors require further investigation to inform better management of fishing activities, maritime traffic, and marine pollution (e.g., aquaculture operations) to mitigate their impact on whale sharks.

Managing stranded large marine animals presents significant challenges in terms of human resources, logistics, and time^26^. For whale sharks, time is crucial during strandings, as they cannot survive long out of water. This study offers important guidance for developing a national plan of action for whale shark conservation in Indonesia by identifying specific locations and seasons that should be prioritized for resource allocation and logistical planning to anticipate strandings, particularly during upwelling periods in areas currently showing an increasing trend (Fig. 4).

Our study also highlights that, in most cases, initial observations of stranded whale sharks in Indonesia were made while they were still alive. With the current success rate of releasing rescued whale sharks back into the sea at 71%, there is room for improvement by strengthening the existing stranding response network in the country. This includes providing teams with adequate training, such as accurate photo identification methods and sex determination techniques for individual whale sharks^38^ and fostering an understanding of the biological differences between marine mammals, especially large whales, and whale sharks to implement distinct and effective stranding response practices^23^. Moreover, conducting outreach and education on whale shark stranding management to local communities in hotspot areas is crucial for effective whale shark stranding management, as local communities play a key role in rapid response efforts^26^.

Lastly, future stranding events should be closely monitored with efforts to rescue the animal. If the animal does not survive, necropsies should be conducted whenever possible to help identify potential contributing stressors. Furthermore, exploring the population connectivity between stranded whale sharks in Indonesia and nearby constellations will provide insights into how these threat levels influence regional populations.

## Methods

### Study area

This study documents whale shark stranding events across Indonesia, an archipelagic nation with the world’s second-longest coastline (108,000 km)^71^. Positioned between the Indian and Pacific Oceans, Indonesia’s waters are shaped by diverse topography. These features support a wide range of habitats, from shallow waters (e.g., the Sunda Shelf and the Arafura Sea) to deep-sea environments (e.g., the Java Trench and the Banda Sea)^72^. Oceanographic features, including upwelling zones^69,73^, thermal fronts^74^, and mesoscale eddies^75^, particularly in deep-sea areas such as the Savu Sea, Banda Sea, and southern coast of Java, play a key role in enhancing marine productivity^69,73^.

Indonesia’s climate is dominated by monsoons, with the southeast monsoon (June–August) and northwest monsoon (December–February) driving seasonal upwelling^69,73^. The southeast monsoon brings nutrient-rich waters to areas like the southern coasts of Java and Nusa Tenggara^66,76^ while the northwest monsoon generates upwelling along the northern coasts of Papua and the Lesser Sunda Islands^69^. These processes create rich feeding grounds that attract whale sharks to Indonesian waters.

### Stranding data

Given that many whale shark stranding reports in Indonesia are published in news media rather than scientific literature, we conducted keyword-based data mining using the Google search engine. To maximize data retrieval, we used a combination of national (Bahasa Indonesia), and English keywords: *hiu paus terdampar*, *hiu tutul terdampar*, *hiu totol terdampar*, *hiu terdampar*, *paus terdampar*, whale shark *terdampar* , whale shark stranded, and whale shark stranding. The search process was conducted over a span of 80 days, from October 2023 to January 2024, and involved approximately 2,000 browsing iterations, continuing until no further unique or relevant information could be found through the search engine.

Stranding records were included based on the following criteria: (1) clear identification of the species as a whale shark, (2) verifiable location within Indonesian waters, and (3) documentation of at least the date and location of the event. Records lacking sufficient species or location information were excluded.

Moreover, we collected as much information as possible, including administrative details (province, regency, district, and village), specific landmarks at the stranding site (e.g., beach names), event date, number and total length of stranded individuals, condition upon first observation, response actions taken, and supporting documentation for validation. Government-sourced data often included precise geographic coordinates, while locations without coordinates were manually georeferenced using nearby identifiable landmarks.

In total, we compiled 119 whale shark stranding records from 2011 to 2023, sourced from peer-reviewed articles (*n* = 10), news media (*n* = 58), social media (*n* = 2), local community reports (*n* = 2), and government records (*n* = 47). Following quality control, four records were excluded due to species misidentification, with three involving baleen whales and one from a misclassified social media post. The final dataset, consisting of 115 validated stranding events, is openly available at 10.5281/zenodo.16731151.

### Analysis of stranding case demographics

To determine whether there are significant differences in whale shark stranding cases or individual counts across provinces, stranding codes, handling responses, size classes, and life stages, a Kruskal-Wallis test was performed, followed by post-hoc Dunn’s test for further analysis. Size classes were determined by grouping sizes in 99 cm increments. For instance, size class two ranged from 200 to 299 cm, and subsequent size classes were grouped similarly. Size classes were used to categorize different life stages, including small juveniles (≥ 1.5 m to < 3 m), large juveniles (≥ 3 m to ≤ 9 m), and adults (> 9 m)^52^. Additionally, the Mann-Whitney U test was used to evaluate the significance of differences between stranded whale sharks that were successfully released into the wild and those that died, as well as to compare stranding categories (single vs. mass strandings). Here, single-stranding events are defined as events with only one stranded animal, and mass-stranding events as events with more than one stranded animal^77^. Annual trends in whale shark stranding cases and individual occurrences were analyzed using linear regression. To evaluate the success rate of whale shark stranding responses, we considered only the total number of alive-stranded cases in each province (*n* = 48) with known final outcomes of handling responses (i.e., released, buried, burned, chopped, drowned, left, or washed away). The success rate was calculated by dividing the number of cases where the whale sharks were successfully released into the wild by the total number of alive-stranded cases in each province. All statistical analyses were conducted using R Studio 2024.09.0^78^.

### Spatial hotspots analysis

We plotted and aggregated stranding data within a radius of 50 km^26,35,79^ using ArcGIS Pro 3.03 (ESRI Inc., Redlands, CA, USA) to investigate the spatial and temporal hotspots of the stranding events. The ‘Optimized Hot Spot’ spatial analyst geoprocessing tool, using Getis-Ord Gi* statistics, identified stranding hotspots over thirteen years for both single cases and individual models. Significant hotspots were detected at three confidence intervals: 90% (*p* < 0.10), 95% (*p* < 0.05), and 99% (*p* < 0.01)^80,81^, using fixed distance bands (100 km^26^). This concept has been widely used in various fields, including biodiversity studies^82^, crime and emergency reporting^83^, deforestation analysis^84^, disease outbreak investigations^85^, and marine mammal stranding research^26,35,79^. In this context, a hotspot refers to an area within a 100 km radius where unusually high numbers of events cluster together, such as a stretch of the coast with many whale shark strandings. Areas that do not meet statistical significance are considered non-significant^26^.

### Spatial and temporal hotspots analysis

Patterns and trends in the annual frequency of stranding events were examined using the ‘Emerging Hot Spot Analysis’ space-time pattern mining geoprocessing tool. This tool integrates both the Getis-Ord Gi* statistics, which can be used to identify the degree of spatial hotspot, and the Mann–Kendall statistics temporal aspects of stranding events (in our case is annual) to determine if there is any annual pattern and trends in each grid of stranding locations. A Network Common Data Form (netCDF) file was formed to create a space-time cube, aggregating the stranding data points throughout Indonesia with a resolution of 50 km and neighborhood distances of 100 km. We use a fishnet grid as an aggregation shape type. This space-time cube assigned latitude and longitude coordinates (X, Y) and the time in a year (Z).

The Emerging Hot Spot Analysis tool, using the Getis-Ord Gi* statistic, identified clustering patterns of high or low whale stranding counts within a space-time cube^86^. It measured the intensity of clustering in a bin relative to its spatial and temporal neighbors, using a fixed spatial distance of 50 km and a temporal interval of 1 year. Statistically significant z-scores (above 1.96 or below − 1.96) indicated hotspots or cold spots at a significance level of *p* < 0.05^87^. Higher z-scores represented more intense hotspots, while lower z-scores signified more substantial cold spots^84^. The null hypothesis assumed no spatial clustering of events, with an expected sum of zero.

Following the Getis-Ord Gi* statistic, the Mann-Kendall trend test was applied to assess temporal trends in the 13-year time series of z-scores for each bin^86^. This test evaluated correlations between consecutive time steps, assigning a value of + 1 for an increasing trend and − 1 for a decreasing trend. The Mann-Kendall statistics and its z-score and p-value were calculated for each bin to determine the significance of temporal trends. The null hypothesis assumed no temporal trend, with an expected sum of zero. Based on variance and the number of time steps, the observed sum was compared to the expected sum to identify statistically significant trends (*p* < 0.05).

The output from this combined spatial–temporal analysis classified each location into hotspot patterns as defined by Dudhat et al. (2022)^79^: consecutive hotspots are locations consistently identified as hotspots across multiple consecutive years, indicating persistent and recurring patterns; sporadic hotspots are locations appearing as hotspots only in certain years, reflecting occasional clustering of events; and new hotspots are locations not previously identified as hotspots but emerging as statistically significant in the most recent year, suggesting shifts in patterns or the emergence of new underlying drivers.

### Oceanographic analysis

Visual inspection suggests that whale shark strandings are associated with high sea surface chlorophyll-a (SSC), maximum wave height (MWH), and low sea surface temperature (SST), particularly during the Southeast Monsoon (SEM) and seasonal transition II periods in the southern coast of Java and Lesser Sunda Ecoregion. Daily and monthly climatological composites for SSC, SST, and MWH from 2018 to 2023 were plotted using Hovmöller diagrams. This approach visually examined spatial and temporal patterns in stranding cases along the western coast of Sumatra, the southern coast of Java, and the Lesser Sunda Seascape as areas of interest (AoI; Fig. 5L) where most cases (88 out of 115) occurred. A 0.5° x 0.5° grid size was used to extract remotely sensed data: SST (1 km resolution) from the Group for High-Resolution Sea Surface Temperature (produced by the Jet Propulsion Laboratory Our Ocean Group^88,89^, SSC (4 km resolution) from the Ocean Colour^90^, and MWH (31 km resolution) from Copernicus Climate Change Service^91^, combining swell and wind waves from ECMWF Reanalysis v5. Daily MWH data represented the highest wave height recorded each day. For investigating the interannual variation of oceanographic parameters and whale shark stranding, we also used Oceanic Nino Index (ONI) from https://origin.cpc.ncep.noaa.gov/products/analysis_monitoring/ensostuff/ONI_v5.php and Dipole Mode Index (DMI) from https://psl.noaa.gov/gcos_wgsp/Timeseries/DMI/.

The influence of seasonal monsoons was analyzed using a Negative Binomial Generalized Additive Model (GAM) in R Studio with the “mgcv” package^92^ to investigate (1) whether seasonal and ecoregion factors significantly impact reported strandings and (2) how variations in SST, SSC, and MHW affect the number of stranded individuals in this region. Model comparisons were performed using the “MuMIn” package with the “pdredge” function, selecting the best model based on the lowest delta second-order Akaike Information Criterion (ΔAICc) value. Models with ΔAICc ≤ 2 were considered top-performing, as a lower ΔAICc indicates stronger explanatory power^93,94^ (Supplementary Table 1).

## Supplementary Information

Below is the link to the electronic supplementary material.

Supplementary Material 1

## Data Availability

The dataset is available at https://doi.org/10.5281/zenodo.16731151. However, data containing latitude and longitude coordinates can be obtained upon request from the corresponding author.
