# Correction: Spatio-temporal patterns, trends, and oceanographic drivers of whale shark strandings in Indonesia

**DOI:** 10.1038/s41598-026-36867-7

**Published:** 2026-01-22

**Authors:** Mochamad Iqbal Herwata Putra, Anindya Wirasatriya, Haidar Asyraffauzan, Fahmi, Ismail Syakurachman, Abdi Hasan, Hanggar Prasetio, Abraham Sianipar, Edy Setyawan, Prabowo Prabowo, Muhammad Subhan Wattiheluw, Arief Edy Handoyo, Muhammad Firdaus Agung Kunto Kurniawan, Mark V. Erdmann, Jatna Supriatna, Masita Dwi Mandini Manessa

**Affiliations:** 1 Department of Geography, Faculty of Mathematics and Natural Sciences, Universitas Indonesia, Depok, Indonesia; 2 Focal Species Conservation Program, Ocean and Science Department, Konservasi Indonesia, Jakarta, Jakarta Indonesia; 3 Department of Oceanography, Faculty of Fisheries and Marine Science, Universitas Diponegoro, Semarang, Indonesia; 4 Center for Coastal Rehabilitation and Disaster Mitigation Studies, Universitas Diponegoro, Semarang, Indonesia; 5 Research Center for Oceanography, National Research and Innovation Agency, Jakarta, Indonesia; 6 Department of Biology, Faculty of Mathematics and Natural Sciences, Universitas Indonesia, Depok, Indonesia; 7 Elasmobranch Institute Indonesia, Denpasar, Bali Indonesia; 8 Directorate of Aquatic Biota and Ecosystem Conservation, Directorate General of Marine Spatial and Ocean Management, Ministry of Marine Affairs and Fisheries, Jakarta, Indonesia; 9 Conservation International Asia-Pacific, Auckland, New Zealand; 10 ReShark, Auckland, New Zealand

Correction to: *Scientific Reports* 10.1038/s41598-025-20543-3, published online 17 October 2025

The original version of this Article contained an error in the spelling of the author Mochamad Iqbal Herwata Putra which was incorrectly given as Mochamad Iqba Herwata Putra.

In addition, in Figure 1B, the percentage of dead whale sharks was incorrect. The original Figure 1 and accompanying legend appear below.

**Fig. 1:**
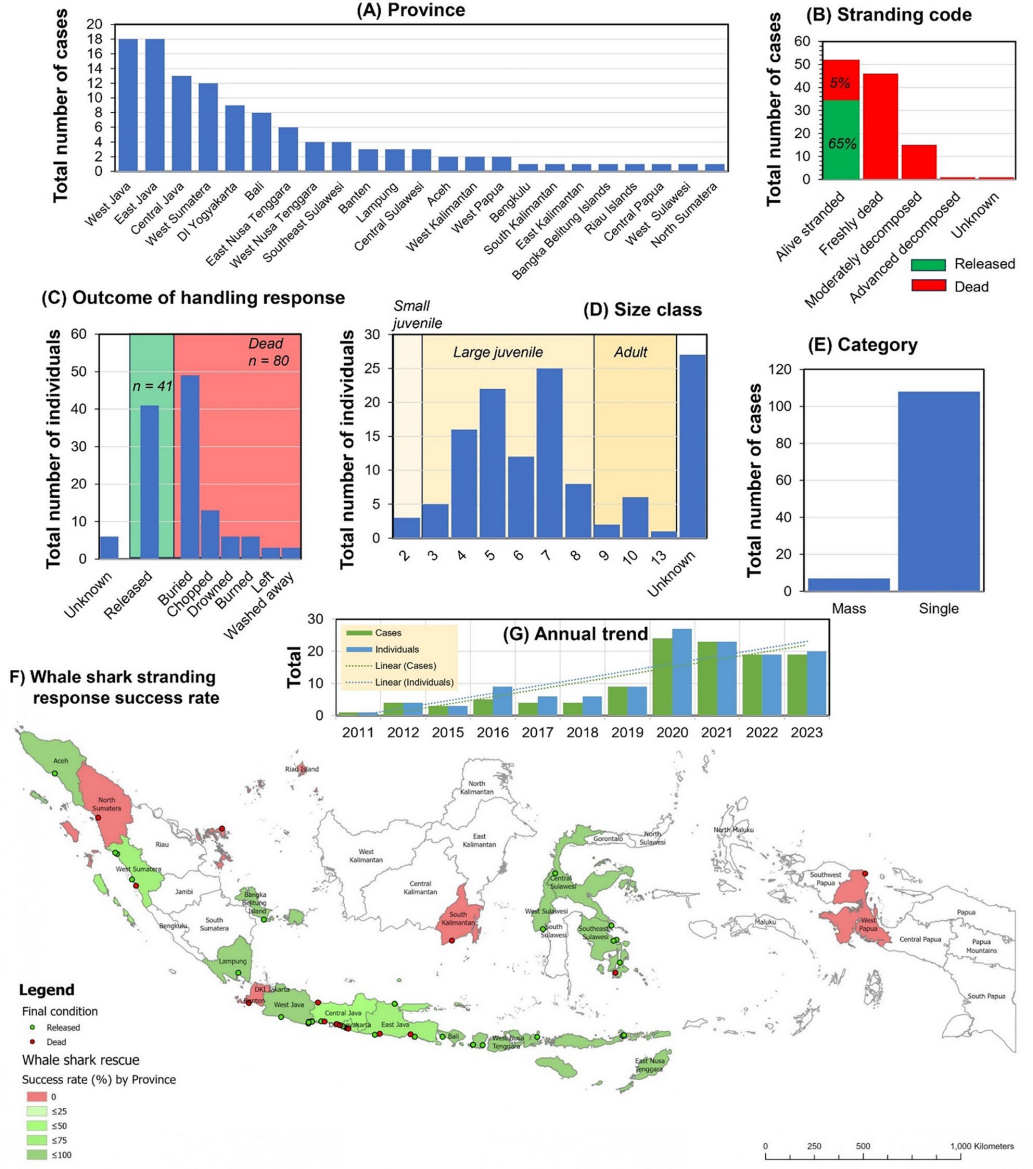
The number and distribution of whale shark stranding cases by province (**A**), condition (**B**), category (**C**), outcome of handling response (**D**), size class distribution (x-axis denotes total length TL in meters, with 3 size classes indicated of small juvenile (< 3 m), large juvenile (3–9 m), and adult (> 9 m); UNK: Unknown (**E**), success rate (summarized by province) of attempted stranding rescues of alive-stranded whale sharks (**F**), and annual trends (**G**).

The original Article has been corrected.

