# Supplementary material for: Spatio-temporal patterns, trends, and oceanographic drivers of whale shark strandings in Indonesia
Source: Sci Rep. 2025 Oct 17;15:36435. doi: 10.1038/s41598-025-20543-3 (PMC12534499; doi:10.1038/s41598-025-20543-3)
Supplement: Supplementary file 1 — Supplementary Material 1 [file 41598_2025_20543_MOESM1_ESM.pdf]

Supplementary Table 1. Negative-binomial generalized additive models were used to describe the seasonal trends of whale shark strandings. ΔAICc represents the difference in AICc between each model and the best-fitting model, with lower ΔAICc values indicating greater explanatory power. The AIC weight (ωi) represents the probability that a model is the best among the sets. The model with the lowest ΔAICc value was selected as the best fit. The symbol "+" denotes the predictors included in the model.

No	(Intercept)	s(Season, bs = "sz", k = 10)	s(SSC, bs = "ts", k = 10)	s(SST, bs = "cc", k = 10)	s(Wave , bs = "cc", k = 10)	df	logLik	AIC	ΔAICc	ωi
16	-0.17	+	+	+	+	15	-58.42	147.71	0.00	0.47
10	-0.15	+			+	10	-64.63	149.76	2.05	0.17
12	-0.15	+	+		+	10	-64.63	149.76	2.05	0.17
14	-0.15	+		+	+	10	-64.63	149.76	2.05	0.17
15	-0.04		+	+	+	12	-65.31	155.05	7.34	0.01
8	0.03	+	+	+		11	-67.90	159.65	11.94	0.00
7	0.06		+	+		9	-70.64	161.25	13.54	0.00
13	0.03			+	+	10	-71.28	162.73	15.02	0.00
11	0.12		+		+	6	-77.14	167.84	20.13	0.00
9	0.15				+	5	-79.67	171.04	23.33	0.00
6	0.14	+		+		9	-76.50	171.14	23.43	0.00
4	0.23	+	+			6	-80.41	174.30	26.59	0.00
5	0.20			+		6	-80.53	174.63	26.92	0.00
2	0.32	+				3	-87.90	183.62	35.91	0.00
3	0.40		+			2	-92.41	188.87	41.16	0.00
1	0.46					1	-96.70	195.39	47.68	0.00

Supplementary Figure 1. Annual whale shark strandings occur across 23 provinces in Indonesia.

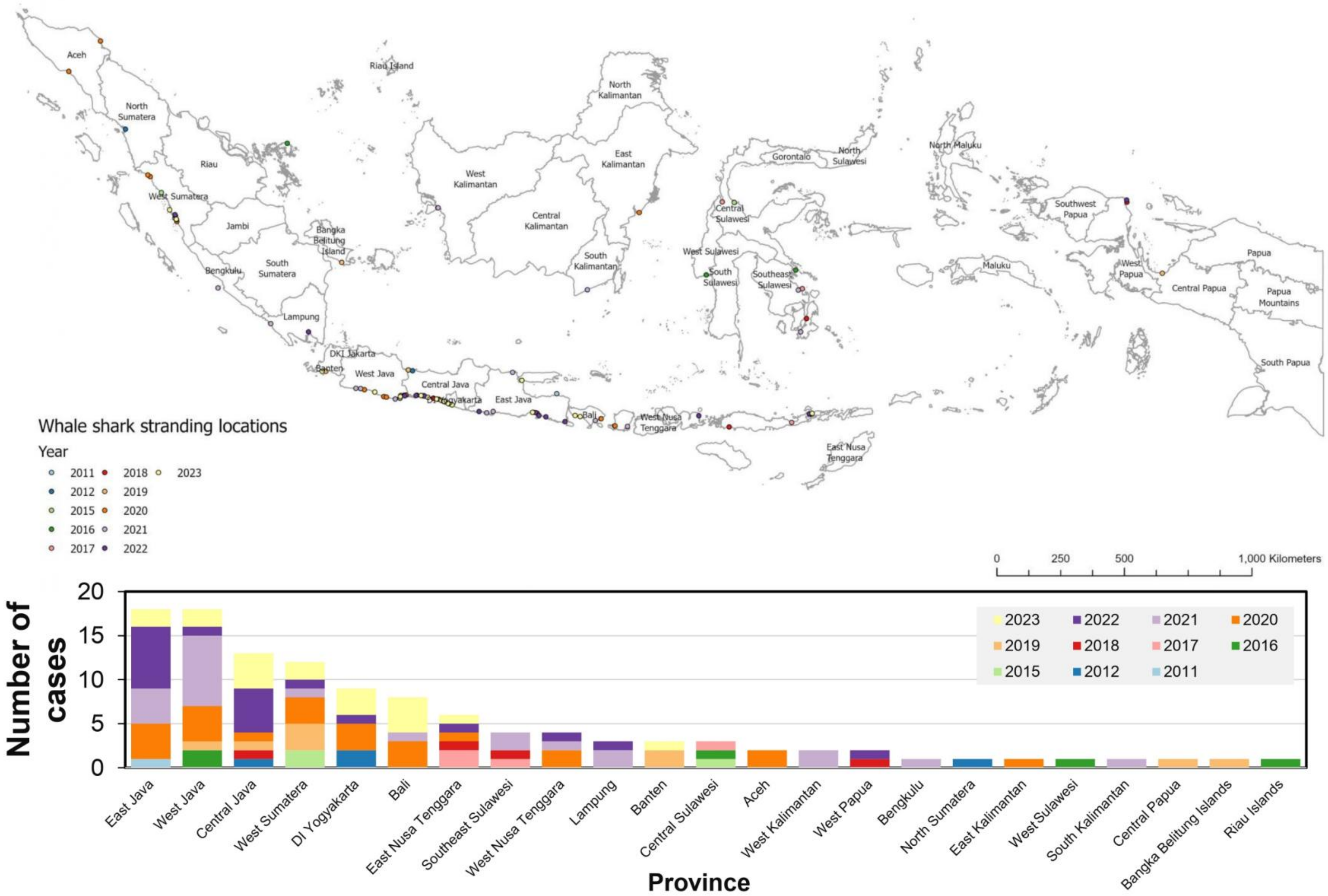

Supplementary Figure 2. Whale shark stranding codes incidents across 23 provinces in Indonesia.

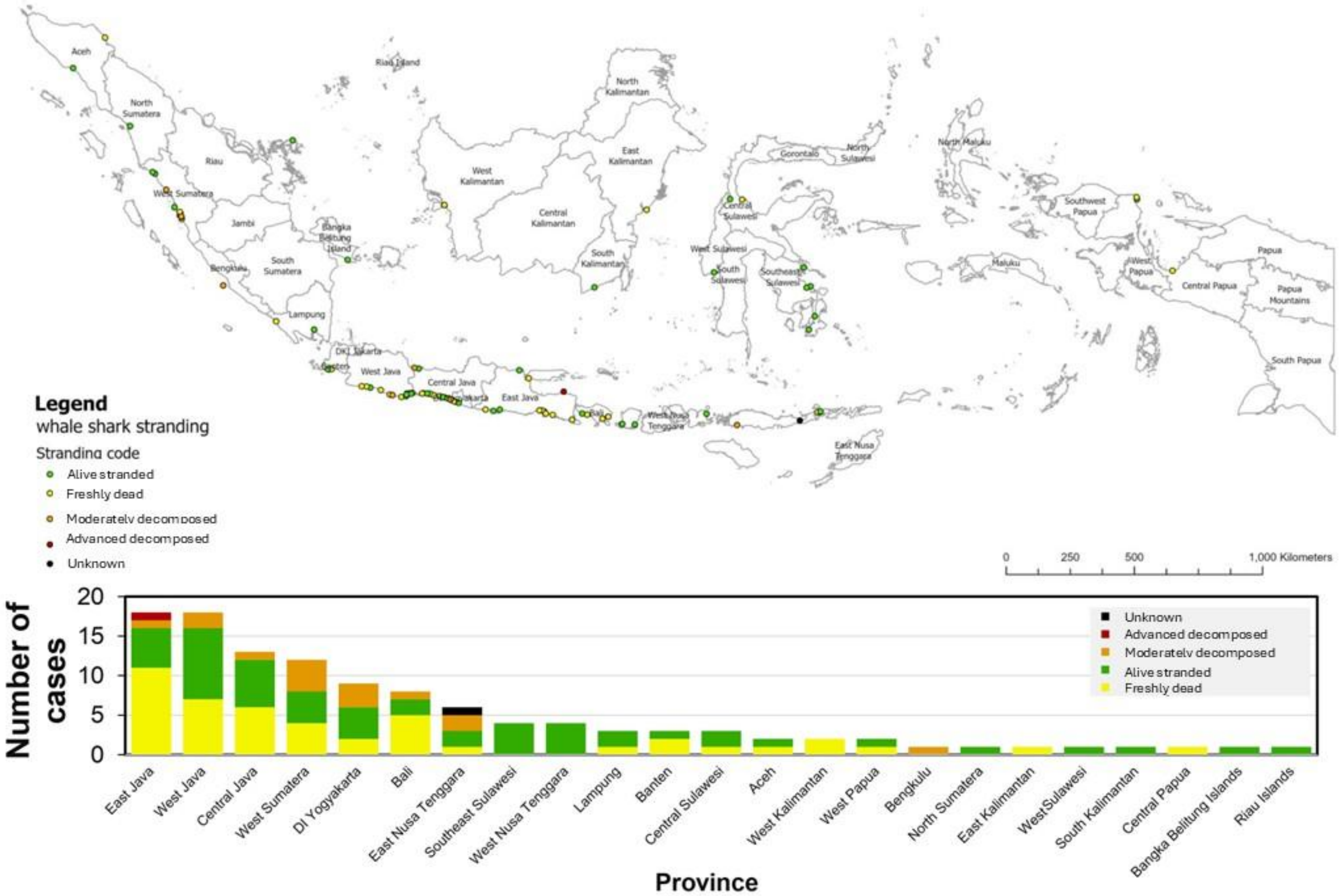

Supplementary Figure 3. Whale shark stranding handling responses from 23 provinces in Indonesia.

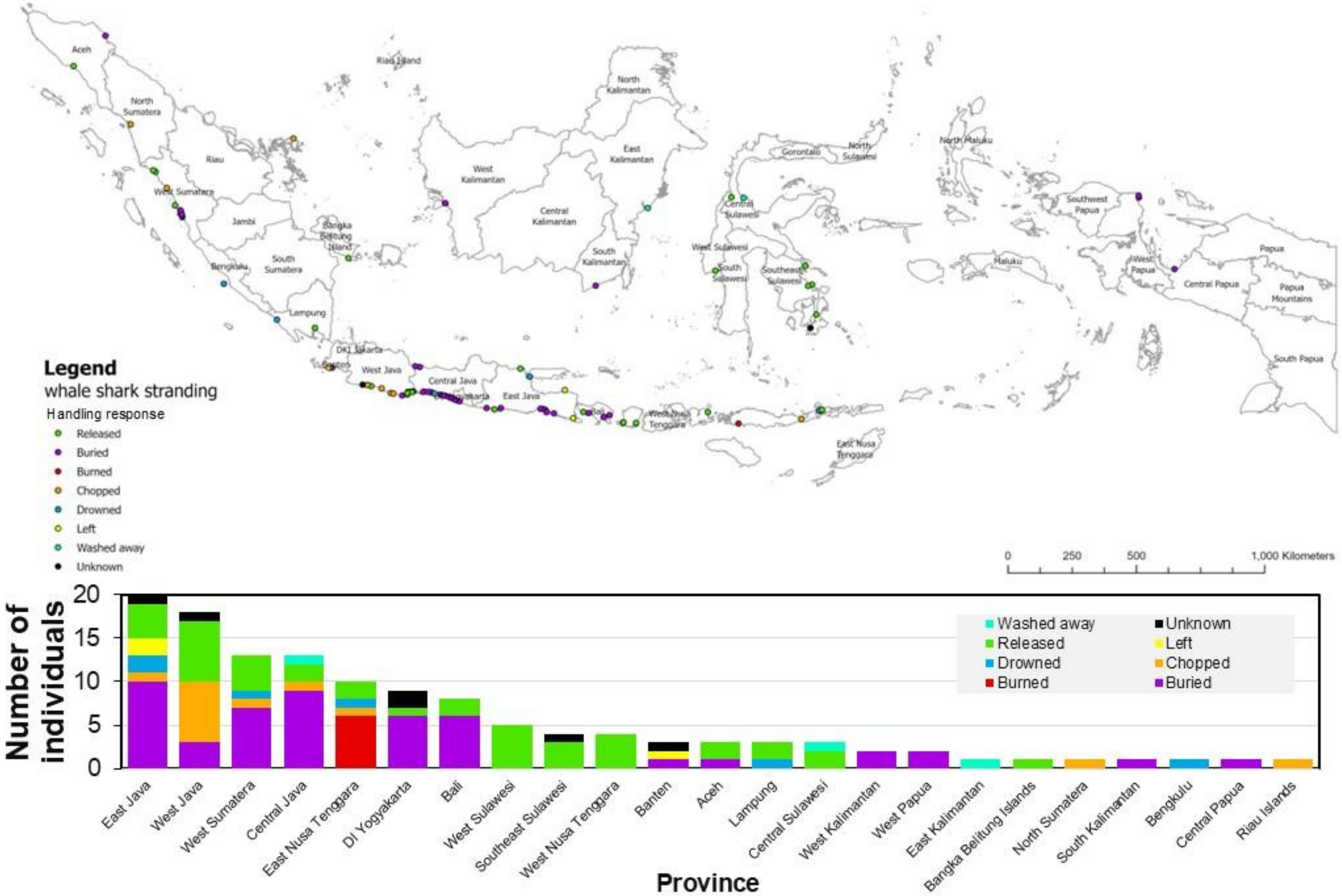

Supplementary Figure 4. Size classes of stranded whale sharks across 23 provinces in Indonesia.

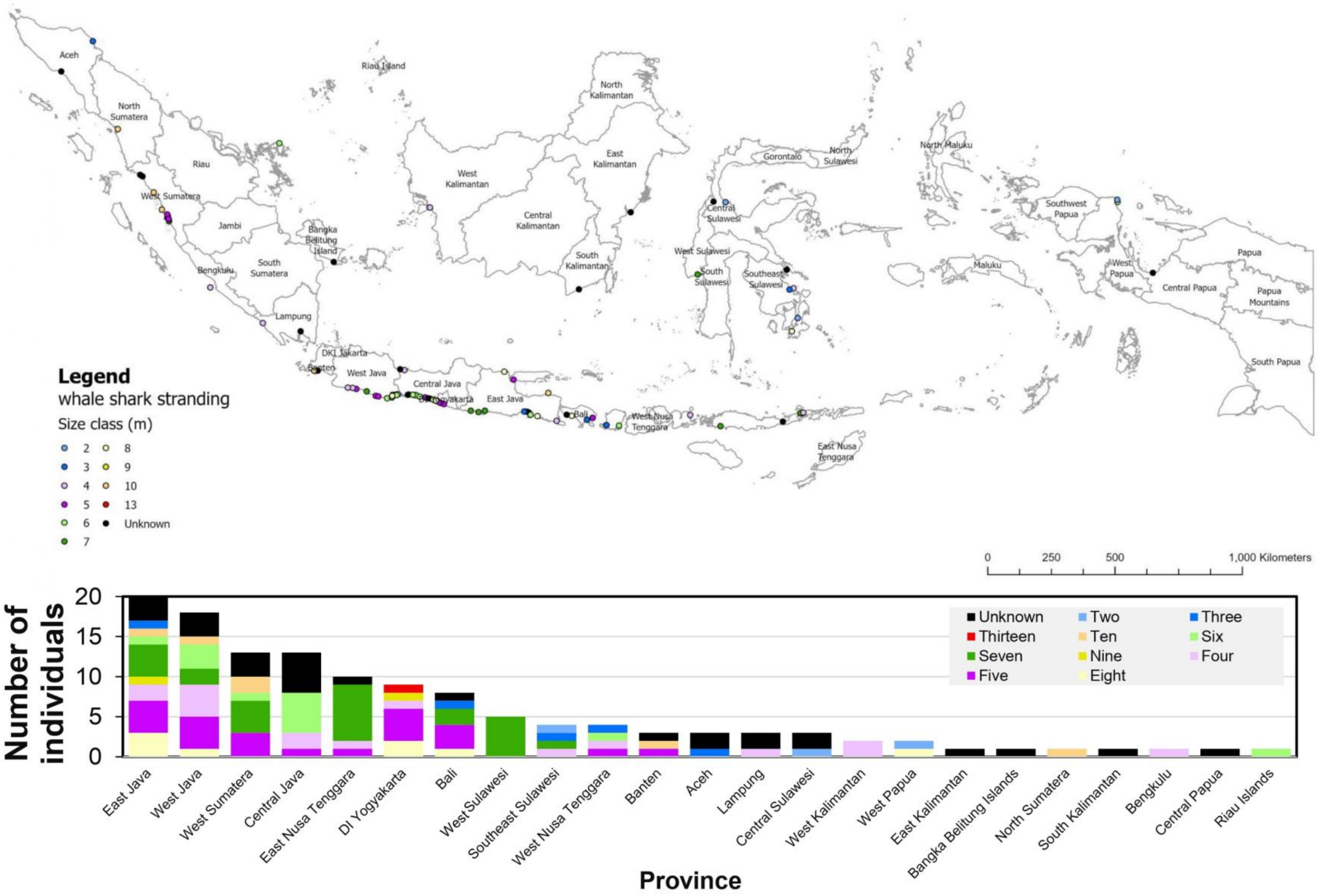

Supplementary Figure 5. Distribution of whale shark stranding categories, including both single and mass stranding cases, across 23 provinces in Indonesia.

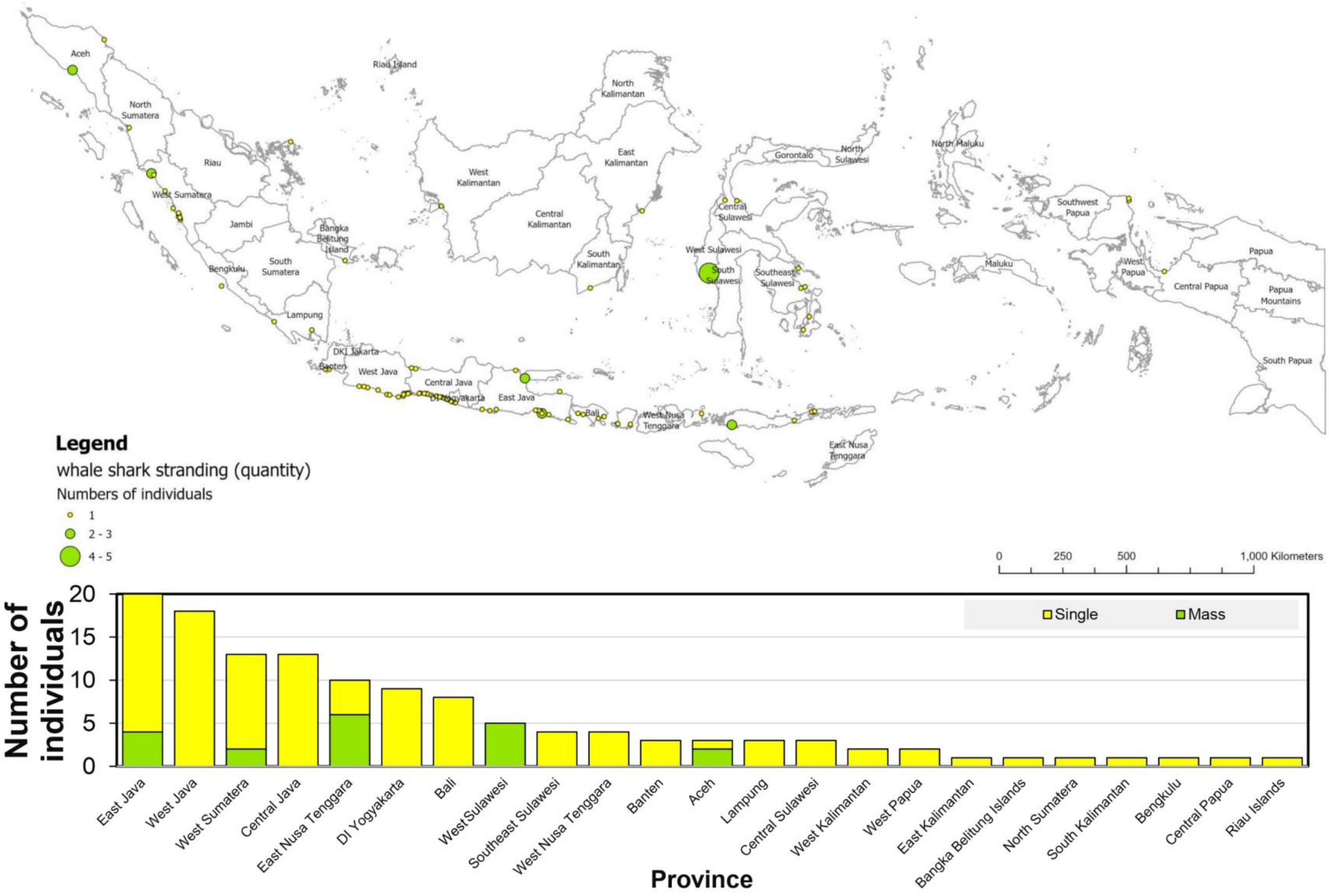

Supplementary Figure 6. Seasonal distributions of whale shark strandings across 23 provinces in Indonesia.

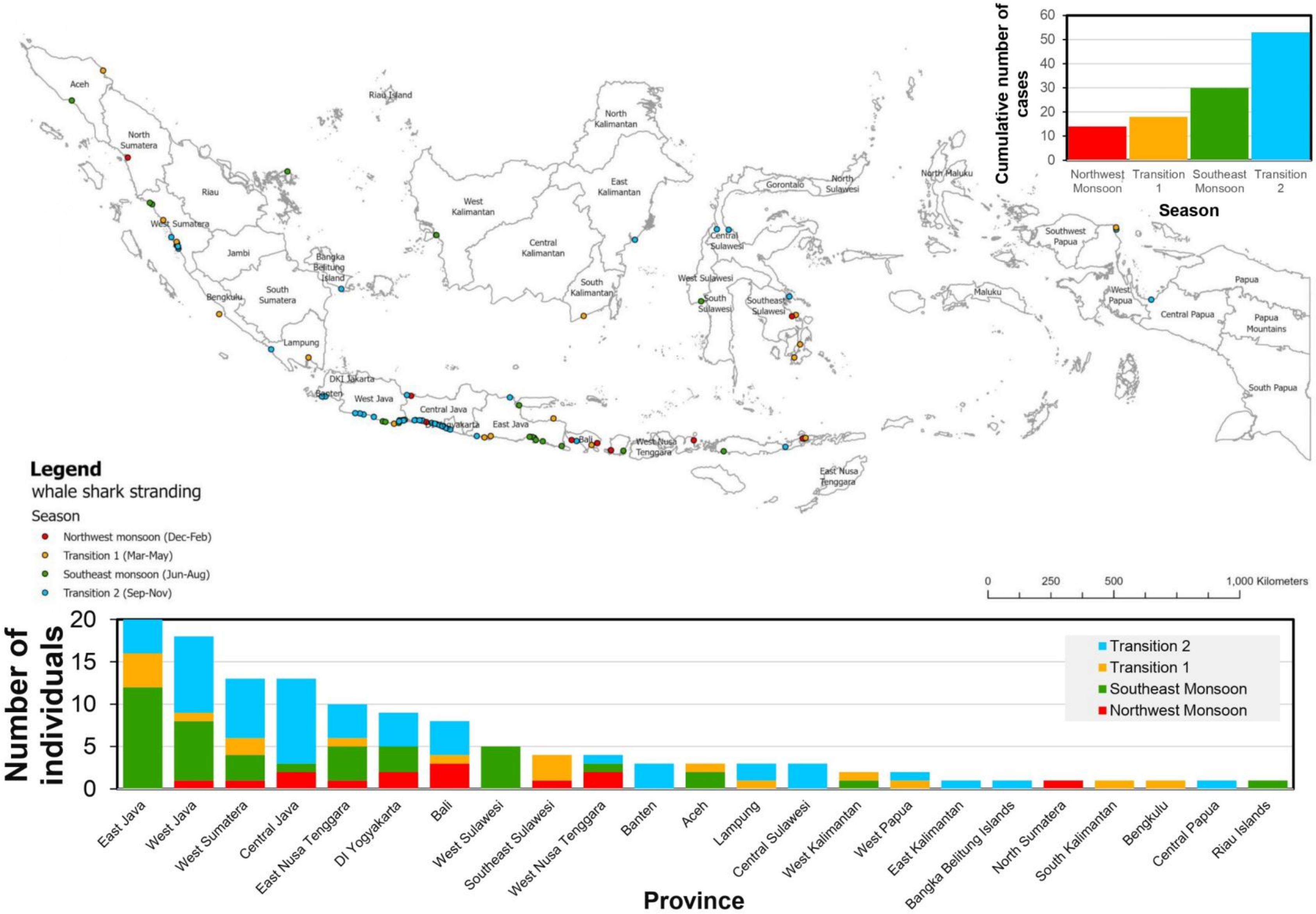

Supplementary Figure 7. (A) Satellite tracks of whale sharks from Ningaloo Reef and Christmas Island (Womersley et al 2022) and Saleh Bay (Setyawan et al, in prep) showing their visits to stranding hotspot areas in the southern Java Sea. (B) Satellite track of a single individual whale shark (total length: 5.60 and sex: male) in Cenderawasih Bay (Setyawan et al, in prep) that stranded itself on 25 September 2019, presumably while chasing anchovies into shallow waters during a period of high productivity (as estimated from monthly chlorophyll-a concentration; data source: Aqua MODIS, 4 km spatial resolution, accessed via https://apdrc.soest.hawaii.edu/erddap/griddap), leading to its stranding. Map created using ArcGIS Pro 3.0.3 (https://pro.arcgis.com/en/pro-app/latest/get-started/download-arcgis-pro.htm).

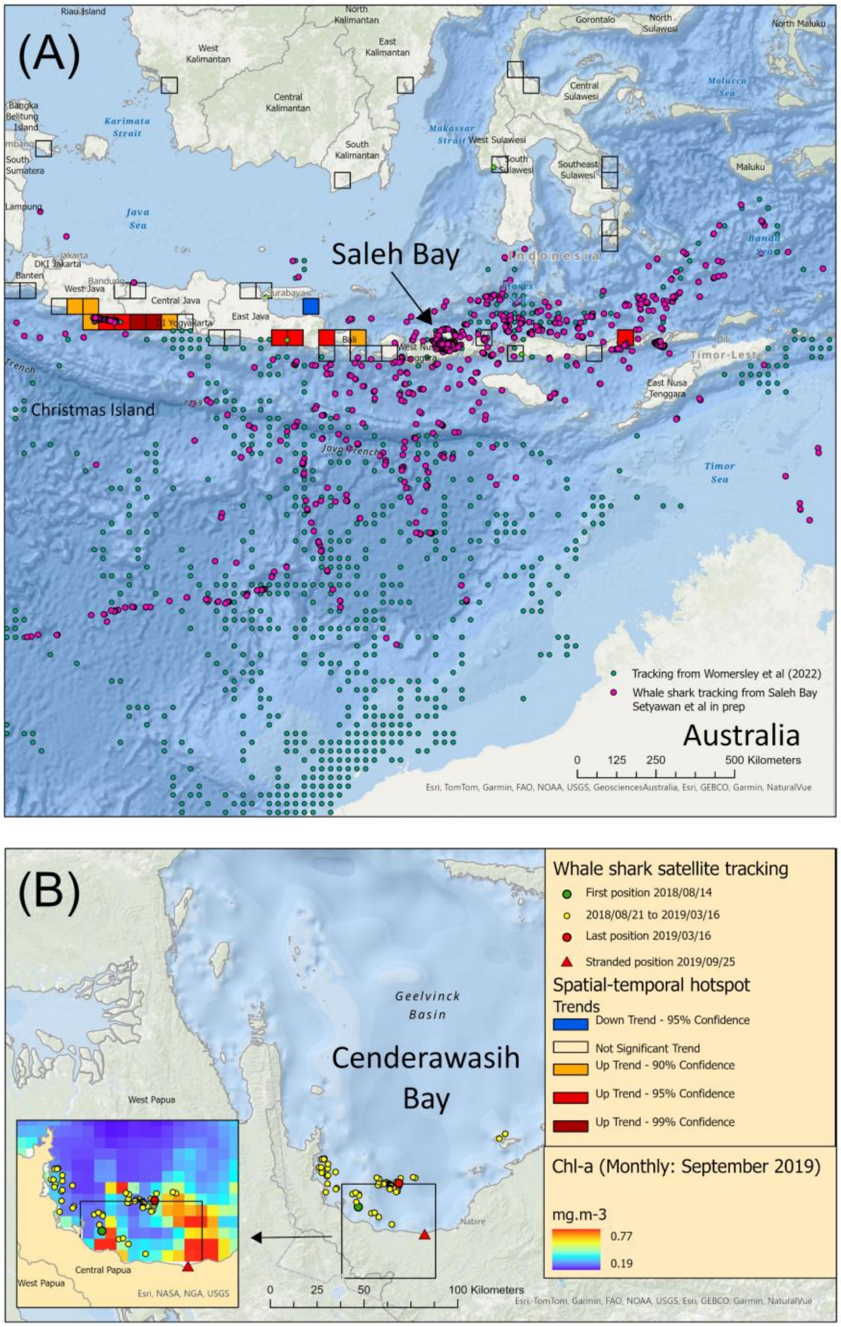

Supplementary Figure 8. Oceanic Niňo Index (ONI) and Dipole Mode Index (DMI) from 2018 to 2023. More than 0.5°C (less than -0.5°C) indicates El Niňo (La Niňa) while more than 0.25°C (less than -0.25°C) indicates positive (negative) IOD event.

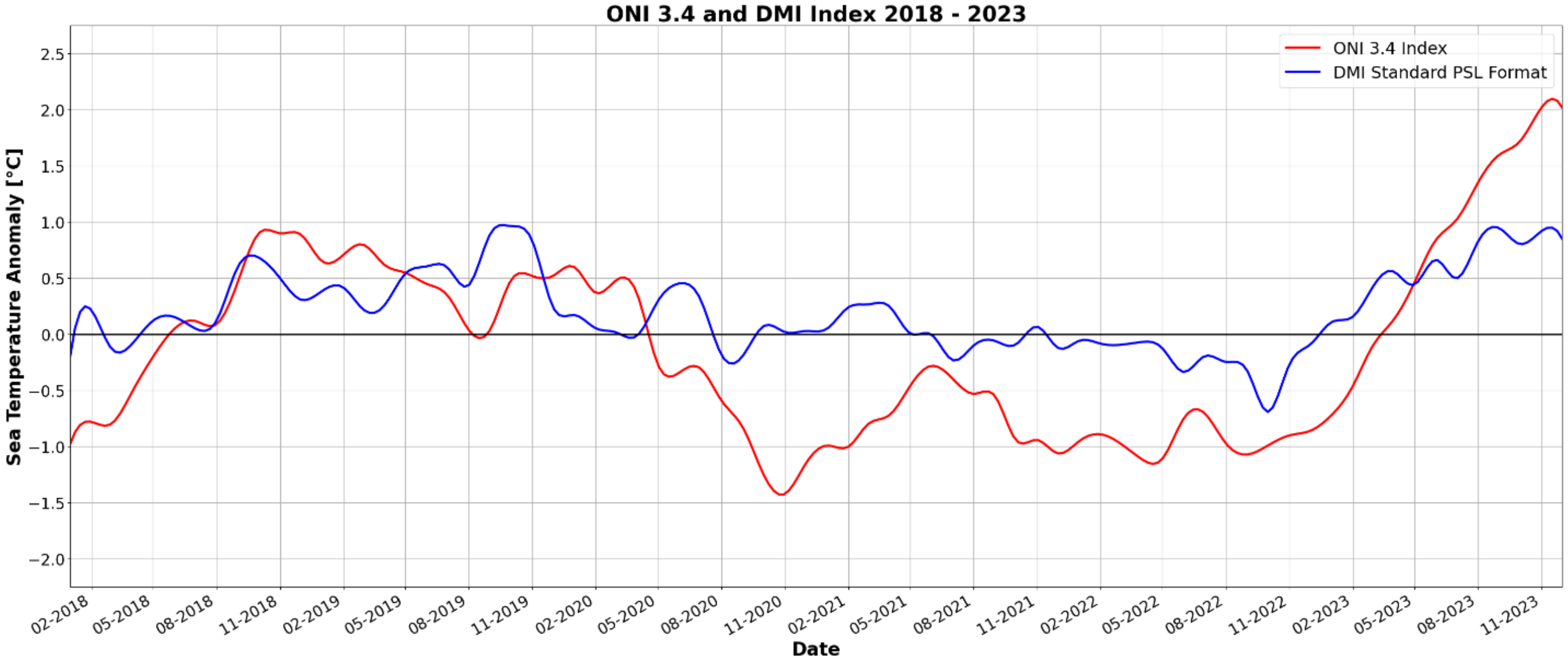

Reference

Womersley, F. C. *et al.*, Global collision-risk hotspots of marine traffic and the world's largest fish, the whale shark. *Proc. Natl. Acad. Sci. USA.* **119** (20), p.e2117440119 (2022).

Setyawan, E. *et al.*, Spatial habitat segregation and residency of whale sharks between the Sunda and Sahul Shelves: Evidence from fin-mount satellite tracking data. (in prep).
